# Short leukocyte telomere length predicts incidence and progression of carotid atherosclerosis in American Indians: The Strong Heart Family Study

**DOI:** 10.18632/aging.100671

**Published:** 2014-05-28

**Authors:** Shufeng Chen, Jue Lin, Tet Matsuguchi, Elizabeth Blackburn, Fawn Yeh, Lyle G. Best, Richard B. Devereux, Elisa T. Lee, Barbara V. Howard, Mary J. Roman, Jinying Zhao

**Affiliations:** ^1^Department of Epidemiology, School of Public Health and Tropical Medicine, Tulane University, New Orleans, LA 70112, USA; ^2^Department of Evidence Based Medicine and Division of Population Genetics, State Key Laboratory of Cardiovascular Disease, Cardiovascular Institute and Fuwai Hospital, National Center for Cardiovascular Diseases, Chinese Academy of Medical Sciences and Peking Union Medical College, Beijing, China; ^3^Department of Biochemistry and Biophysics, University of California, San Francisco, CA 94143, USA; ^4^Center for American Indian Health Research, University of Oklahoma Health Science Center, Oklahoma City, OK 73104, USA; ^5^Missouri Breaks Industries Research Inc, Timber Lake, SD 57656, USA; ^6^Weill Cornell Medical College, New York, NY 10065, USA; ^7^Medstar Research Institute and Georgetown and Howard Universities Centers for Translational Sciences, Washington, DC 20007, USA

**Keywords:** leukocyte telomere length, carotid atherosclerosis, risk prediction, American Indians, Strong Heart Study

## Abstract

Short leukocyte telomere length (LTL) has been associated with atherosclerosis in cross-sectional studies, but the prospective relationship between telomere shortening and risk of developing carotid atherosclerosis has not been well-established. This study examines whether LTL at baseline predicts incidence and progression of carotid atherosclerosis in American Indians in the Strong Heart Study. The analysis included 2,819 participants who were free of overt cardiovascular disease at baseline (2001-2003) and were followed through the end of 2006-2009 (average 5.5-yr follow-up). Discrete atherosclerotic plaque was defined as focal protrusion with an arterial wall thickness ≥50% the surrounding wall. Carotid progression was defined as having a higher plaque score at the end of study follow-up compared to baseline. Associations of LTL with incidence and progression of carotid plaque were examined using Cox proportional hazard regression, adjusting for standard coronary risk factors. Compared to participants in the highest LTL tertile, those in the lowest tertile had significantly elevated risk for both incident plaque (HR, 1.49; 95% CI, 1.09–2.03) and plaque progression (HR, 1.61; 95% CI, 1.26–2.07). Our results provide initial evidence for a potential prognostic utility of LTL in risk prediction for atherosclerosis.

## INTRODUCTION

Telomeres are special chromatin structures and associated proteins at the end of each chromosome that protect chromosomes from degradation and recombination. They are formed by tandem repeats made up of TTAGGG sequence in vertebrates [1]. Telomere length progressively shortens with each cell division to a critical length, called the Hayflick limit, [2] beyond which cell enters senescent state, resulting in a cascade of negative biological processes. Shorter leukocyte telomere length (LTL) has been associated with a variety of age-related disorders, such as atherosclerotic cardiovascular diseases (CVD), [3] diabetes, [4] and cancer [5].

Atherosclerosis is an age-related disorder characterized by atherosclerotic plaque with variable arterial wall thickening. The atherosclerotic plaques comprise inflammatory cells, vascular smooth muscle cells, their secreted products, and intracellular and extracellular lipids. Increased number of senescent cells has been observed in vascular smooth muscle cells, endothelial cells and macrophages in aged arteries and atherosclerotic plaque [6-8]. Cross-sectional epidemiological studies have demonstrated an association of shorter telomere length with atherosclerosis and atherosclerotic CVD, [3, 9-12] but results were inconsistent across studies [13]. Among the very few longitudinal studies, shortened LTL was associated with all-cause mortality in patients with stable coronary artery disease [14] or type 1 diabetes [15], but no study has examined the potential role of telomere shortening in development and progression of carotid atherosclerosis in a large, community-based cohort. The current study seeks to investigate whether LTL could be a potential prognostic factor predicting progression of carotid atherosclerosis in a prospectively examined population of American Indians participating in the Strong Heart Family Study (SHFS).

## RESULTS

### Characteristics of study participants at baseline and end of follow-up

LTL was significantly and inversely correlated with age (*r* = −0.33, P<0.0001). Women had significantly longer LTL than men after adjusting for age (1.02±0.01 vs. 1.00±0.01, age-adjusted P=0.03). Of the 2,819 individuals with no overt CVD at baseline, 544 exhibited plaque progression after a mean follow-up period of 5.4 years, among whom 357 subjects (141 men, 216 women) developed new plaque and 187 (70 men, 117 women) had higher plaque score compared to baseline. Subjects with plaque progression had shorter LTL than those without (0.95±0.20 vs. 1.00±0.23, P<0.001).

Table 1 shows clinical characteristics of study participants at baseline. Age- and sex-adjusted correlations of LTL with traditional coronary risk factors are presented in Table 2. After adjustments for age and sex, LTL was significantly correlated with BMI (*r* = −0.13, P<0.0001) and fasting glucose (*r* = −0.07, P = 0.03), but not other listed covariates.

**Table 1:** Baseline characteristics of study participants with no prevalent CVD (*n*=2,819)

Variables	Mean ± SD or *n* (%)
Leukocyte telomere length	0.99± 0.23
Age, years	38.5±15.8
Men, *n* (%)	1056 (37.5%)
Body mass index, kg/m^2^	32.4±7.9
Systolic blood pressure, mmHg	121.6±16.0
Diastolic blood pressure, mmHg	76.3±11.0
Total cholesterol, mg/dl	180.6±36.9
Triglyceride, mg/dl	164.1±135.4
Low-density lipoprotein cholesterol, mg/dl	98.1±29.0
High-density lipoprotein cholesterol, mg/dl	51.0±14.4
Fasting glucose, mg/dl	113.1±52.1
Estimated glomerular filtration rate, ml/min/1.73m^2^	102.1±26.7
Current smoking, n (%)	971 (34.5)
Current drinking, n (%)	1661 (59.1)
Hypertension, n (%)	841 (29.9)
Diabetes, n (%)	586 (20.8)
Prevalent carotid plaque, n (%)	728 (25.8)
Plaque progression, n (%)	544 (19.3)
Incident carotid plaque, n (%)	357 (17.1)

**Table 2:** Clinical correlates of LTL among participants with no prevalent CVD at baseline (*n*=2,819)

Variables	Correlation coefficient**	P value*
Body mass index	−0.13	<0.0001
Systolic blood pressure	0.04	0.4
Diastolic blood pressure	0.01	0.8
Total cholesterol	−0.03	0.2
Triglyceride	−0.04	0.06
Low-density lipoprotein cholesterol	−0.02	0.2
High-density lipoprotein cholesterol	0.03	0.06
Fasting glucose	−0.07	0.03
Estimated glomerular filtration rate	−0.03	0.6
Current smoking*	-	0.6
Current drinking*	-	0.3

** Adjusted for age and sex;

* P-values by generalized estimating equation to account for family relatedness.

Table 3 lists baseline characteristics of study participants according to LTL tertiles. Compared to subjects in the highest tertile of LTL (longest telomere length), those in the lowest tertile were significantly older, had higher mean BMI, total cholesterol, triglyceride and low-density lipoprotein cholesterol. Prevalence of diabetes was significantly higher among subjects with shorter LTL than those with longer LTL (age-adjusted P trend 0.0004).

**Table 3:** Characteristics of study participants according to LTL tertiles (*n*=2,819)

Variables	Tertile 1 (N=939)	Tertile 2 (N=940)	Tertile 3 (N=940)	P value*
Age, years	44.5±15.3	39.1±15.2	32.0±14.3	<0.0001
Men, *n* (%)	345 (36.7)	366 (38.9)	345 (36.7)	0.9
LTL (T/S ratio)				
Mean	0.75±0.12	0.98±0.05	1.23±0.15	<0.0001**
Median	0.78	0.98	1.19	
Range	0.28-0.90	0.90-1.07	1.07-2.22	
Interquartile range	0.18	0.08	0.16	
Follow-up, years	5.1±1.0	5.5±1.1	5.6±1.1	0.1**
Prevalent plaque, n (%)	345 (36.7)	236 (25.1)	147 (15.6)	0.2**
Plaque progression, n (%)	221 (23.5)	198 (21.1)	125 (13.3)	0.07**
Incident plaque, n (%)	122 (20.5)	141 (20.0)	94 (11.9)	0.9**
Body mass index, kg/m^2^	33.6±8.1	32.5±7.9	31.1±7.5	0.0002**
SBP, mmHg	123.4±16.7	122.3±16.3	119.1±14.6	0.8**
DBP, mmHg	76.8±10.4	76.8±11.6	75.4±10.9	0.2**
Total cholesterol, mg/dl	184.1±36.8	183.1±36.6	174.6±36.6	0.02**
Triglyceride, mg/dl	173.8±141.7	169.5±129.0	149.0±134.2	0.05**
LDL-C, mg/dl	99.5±29.6	100.2±29.3	94.5±27.7	0.02**
HDL-C, mg/dl	51.2±14.1	50.5±14.8	51.1±14.4	0.1**
Fasting glucose, mg/dl	121.7±58.6	113.4±51.6	104.2±43.7	0.06**
eGFR, ml/min/1.73m^2^	97.9±28.0	101.7±26.5	106.7±25.0	0.5**
Current smoking, n (%)	305 (32.6)	329 (35.0)	337 (35.9)	0.8**
Current drinking, n (%)	527 (56.4)	539 (57.5)	595 (63.3)	0.1**
Hypertension, n (%)	347 (37.0)	298 (31.8)	196 (20.9)	0.4**
Diabetes, n (%)	281 (30.0)	194 (20.6)	111 (11.8)	<0.0001**

* Correction for family relatedness by generalized estimating equation;

** Additionally adjusted for age at baseline.

Abbreviations: LTL, leukocyte telomere length; T/S ratio, telomeric product (T)/single copy gene (S) product; eGFR, estimated glomerular filtration rate.

### Prospective association of LTL with incidence and progression of carotid plaque

LTL was significantly associated with incidence and progression of carotid atherosclerosis, independent of potential confounders. In multivariate survival analyses that treated LTL as a continuous variable, the hazards for incidence and progression of carotid plaque were 0.48 (95% CI, 0.27-0.85) and 0.50 (95% CI, 0.32–0.81), respectively. In statistical analyses using LTL tertiles, subjects with shorter LTL (lowest tertile) were significantly more likely to develop new plaque (HR, 1.49, 95% CI, 1.09–2.03) or plaque progression (HR, 1.61; 95% CI, 1.26–2.07) compared to those with longer LTL (highest tertile). Multivariate-adjusted HRs and 95% CIs for incident plaque and plaque progression are shown in Table 4. Kaplan-Meier survival curves for incident plaque are plotted in Figure S1.

**Table 4:** Prospective association of LTL with incidence and progression of carotid plaque in American Indians participating in the Strong Heart Family Study

LTL	HR (95% CI)	P value
**Incident plaque (N=2,091)**		
**Model 1**		
Continuous LTL	0.43 (0.24−0.75)	0.003
Tertile 1 vs. Tertile 3	1.58 (1.16− 2.15)	0.004
Tertile 2 vs. Tertile 3	1.19 (0.88 − 1.60)	0.25
**Model 2**		
Continuous LTL	0.42 (0.24− 0.75)	0.003
Tertile 1 vs. Tertile 3	1.57 (1.15− 2.14)	0.004
Tertile 2 vs. Tertile 3	1.18 (0.88 − 1.59)	0.28
**Model 3**		
Continuous LTL	0.48 (0.27− 0.85)	0.01
Tertile 1 vs. Tertile 3	1.49 (1.09− 2.03)	0.01
Tertile 2 vs. Tertile 3	1.14 (0.85 − 1.54)	0.39
**Plaque progression (N=2,819)**		
**Model 1**		
Continuous LTL	0.45 (0.28 − 0.71)	0.0006
Tertile 1 vs. Tertile 3	1.72 (1.34 − 2.21)	<0.0001
Tertile 2 vs. Tertile 3	1.34 (1.06 − 1.69)	0.01
**Model 2**		
Continuous LTL	0.47 (0.30− 0.75)	0.001
Tertile 1 vs. Tertile 3	1.67 (1.31− 2.14)	<0.0001
Tertile 2 vs. Tertile 3	1.30 (1.03 − 1.64)	0.03
**Model 3**		
Continuous LTL	0.50 (0.32− 0.81)	0.004
Tertile 1 vs. Tertile 3	1.61 (1.26− 2.07)	0.0002
Tertile 2 vs. Tertile 3	1.22 (0.96 − 1.54)	0.10

Model 1: adjusted for age at baseline, sex and study center

Model 2: additionally adjusted for BMI, current smoking and alcohol drinking status

Model 3: further adjusted for diabetes, systolic blood pressure, low-density lipoprotein cholesterol, high-density lipoprotein cholesterol, and estimated glomerular filtration rate

### Results of sensitivity analyses

Sensitivity analyses for incidence and progression of carotid plaque using PROC LIFEREG are shown in Table S1. Results show that additionally taking into account interval-censoring of the data did not change our results. After excluding participants with prevalent diabetes, the associations of LTL with incident plaque (HR, 1.62; 95% CI, 1.14–2.31) and plaque progression (HR, 1.69; 95% CI, 1.27–2.25) remained statistically significant (Table S2). Further exclusion of participants with hypertension did not change our results (Table S3). In addition, results in LTL quartiles were similar to those in tertiles.

## DISCUSSION

In a large, well-characterized prospective cohort of American Indians, we found that LTL significantly predicts incidence and progression of carotid atherosclerosis, independent of established cardiovascular risk factors including diabetes and hypertension. Subjects with shorter LTL have significantly elevated risk of developing carotid atherosclerosis than those with longer telomeres, indicating that LTL could be a valuable prognostic marker for carotid atherosclerosis. Our study provides initial evidence for a prospective association of LTL with carotid atherosclerosis.

Previous studies have examined the association of LTL with subclinical atherosclerosis, [13, 16-18] most of which focused on cross-sectional analyses of ultrasonic measures in subgroups of patients or research subjects. For example, among men participating in the Framingham Heart Study, age-adjusted LTL was significantly associated with internal carotid artery intima-media thickness (IMT) in obese (BMI > 30 kg/m^2^), but not in non-obese men [18]. Another study reported negative association of LTL with common carotid IMT in subjects over 40 years old. [11] Shorter LTL was also associated with carotid artery plaque in hypertensive men [19] or elderly subjects (mean age 73 years old) [17]. In addition, LTL was negatively associated with coronary artery calcification in a low risk cohort free of prevalent CVD [20] and in an urban Arab population [21]. However, other studies did not find an association of LTL with subclinical atherosclerosis as measured by IMT [13]. Moreover, to our knowledge, the prospective association of LTL with incidence or progression of carotid plaque has not been previously examined.

In contrast to results regarding plaque progression, shorter LTL did not predict atherosclerotic progression assessed by an increase in IMT (data not shown) in our study population. Although IMT and plaque are highly correlated, [22] they may reflect different aspects of atherogenesis with distinctive relations to clinical outcomes [23, 24]. In general, IMT is considered as a measure of diffuse or early atherosclerosis as well as arteriosclerosis, whereas plaque is a direct manifestation of atherosclerosis that represents later stage of atherosclerosis more closely related to clinical events. The pathological processes leading to thickening of intima media and to plaque formation may also be different. [25, 26]. In the SHFS, we measured carotid IMT in regions free of plaque, which is different from many previous studies that measured carotid artery wall thickness regardless of the absence or presence of plaque. [27, 28]. In previous studies, we have reported different heritability of subclinical atherosclerosis in the SHFS, [29] suggesting potential differences in genetic and/or environmental mechanisms influencing inter-individual variability in IMT and plaque. We also found that carotid plaque, but not IMT, significantly predicts CVD events in participants of the SHS, [24] further highlighting the potential differences in biological mechanisms leading to intima-media thickening and plaque formation between vascular beds. In a recent study, shorter LTL predicts advanced, but not early, atherogenesis, consistent with our findings of association between telomere shortening and discrete plaque but not diffuse IMT [30]. Therefore, the differential effect of LTL on atherosclerosis as measured by carotid plaque, but not IMT, observed in our study is in agreement with previous findings and suggests that carotid IMT and plaque merit separate analysis in future research.

Our study has several limitations. First, we measured LTL using a PCR-based assay that does not quantify absolute telomere length; hence we were unable to examine the differences in absolute telomere length. However, the qPCR method requires much less amount of DNA than Southern blot and thus is well suited for large-scale epidemiological studies. Moreover, telomere lengths measured by these two methods are highly correlated [31]. Second, telomere lengths may vary among cells in the same tissue and among chromosomes in the same cell [32]. In this study, we only measured telomere length in blood leukocytes but not carotid plaque; however, obtaining plaque tissue is clinically impractical for large-scale epidemiological studies. In addition, previous studies have demonstrated that telomere length in different tissues may be highly correlated [33]. Moreover, the current study does not explain the mechanisms underlying the link between shortened telomere length and development of carotid atherosclerosis. Third, our findings were derived from a cohort of American Indians who suffer from a high rate of diabetes. However, all our statistical analyses adjusted for diabetes status, and excluding participants with diabetes did not change our results. In addition, diabetes prevalence is rising in many other populations, suggesting that our findings may be replicated in other settings. Fourth, although we were able to control many of the potential confounding variables, residual confounding by other factors including time-dependent factors cannot be entirely excluded. Finally, the prospective association of LTL with risk of carotid atherosclerosis identified in our study may not necessarily be causal because baseline factors influencing atherosclerosis, either unmeasured or imperfectly measured, may influence this apparently "causal" relationship.

In summary, shorter LTL significantly predicts increased risk of atherosclerosis development among participants in the SHFS, independent of known coronary risk factors. Our finding provides novel insights into the understanding of biological aging and its role in atherogenesis, which will potentially lead to novel therapeutic options of anti-aging to prevent atherosclerosis. Confirmation in individuals of other ethnic origins with different risk profiles or environment is warranted in future research.

## METHODS

### Study population

The Strong Heart Study (SHS) is a longitudinal study of CVD and its risk factors among 13 American Indian communities residing in three geographic regions in Arizona, Oklahoma, and North and South Dakota. The Strong Heart Family Study (SHFS), a component of the SHS, was initiated in 2001-2003 by recruiting 3,665 tribal members (aged 14-93 years old) from 94 multigenerational families. Detailed study design and methods of the SHS have been described elsewhere. [34, 35] The SHS protocols were approved by the Institutional Review Boards of the Indian Health Service, the participating institutions, and the participating tribes. Written informed consent was obtained from each study participant.

All SHFS participants underwent carotid ultrasound examinations at baseline [2001-2003] and again after 5 years follow-up (2006-2009, 93% of surviving participants). The current analyses of plaque progression included 2,819 participants (1,056 men, 1,763 women) free of overt CVD at baseline. For incident carotid plaque, we further excluded participants with prevalent carotid plaque (n=728), leaving a final sample size of 2,091 (788 men, 1,303 women) for the incidence analyses.

### Carotid ultrasonography

All SHFS study participants underwent carotid ultrasonography using Acuson Sequoia machines equipped with 7 MHz vascular probes on the day of the clinic visit using a standardized protocol as described previously. [24, 26] In brief, with the subject in the supine position, carotid arteries were extensively scanned for atherosclerotic plaque. The presence of discrete atherosclerotic plaque was defined as the presence of focal thickening at least 50% the surrounding wall, which was defined as the uninvolved intimal-medial thickness (IMT) adjacent to the plaque. This is a standard definition used in numerous epidemiologic studies, including the Atherosclerosis Risk in Communities Study (ARIC) [36]. Plaque score, a semi-quantitativemeasure of the extent of atherosclerosis,was calculated by thenumber of left and right segments (common carotid, bulb, internalcarotid, external carotid) containing plaque; thus plaque scoreranged from 0 to 8. [24] Incident plaque was identified if plaque was absent at baseline but plaque was detected at the end of study follow-up. Progression of carotid plaque was defined as a higher plaque score at follow-up compared to baseline.All ultrasound measurements were performed by trained research sonographers and interpreted by a single highly experienced cardiologist (Mary J. Roman) who was blinded to the clinical characteristics of study participants.

### Measurement of leukocyte telomere length (LTL)

LTL was measured by quantitative polymerase chain reaction (qPCR) in Dr. Blackburn's laboratory at UCSF. Detailed laboratory protocol and quality control procedures have been described previously [37-40]. Briefly, LTL was quantified by qPCR using a serially diluted standard DNA and the standard curve method. The ratio of the telomeric product vs. the single copy gene reflects the average length of the telomeres. A single copy gene was amplified in parallel to normalize the quantity of the input DNA. Each DNA sample was assayed three times and the mean value was used in statistical analysis. *For quality control, we included seven* control DNA samples from various cancer cell lines in each assay plate. These control samples allowed us to create standard curves, which were then integrated into a composite standard curve used for T and S concentration calculations. In addition, 4.1% *of the total sample was assayed in duplicate LTL of replicate samples were highly correlated* (*r* = 0.95, p<0.0001). Theintra- and inter-assay %CV was 4.6% and *6.9%, respectively*. Lab technicians were blinded to any knowledge of clinical data.

### Assessments of risk factors

The SHS consisted of a personal interview, a physical examination, and laboratory tests. The personal interview collected information for demographics, medical history, and lifestyle factors including smoking, alcohol intake, habitual diet and physical activity. The physical examination included anthropometric measures, blood pressure measurements and an examination of the heart and lungs. Fasting blood samples were collected to measure lipids, glucose, insulin, and inflammatory biomarkers. Urinary albumin and creatinine was measured in a spot urine sample collected on the day of clinical visit. Details of the study protocol and lab methods have been described previously. [34]

Current smoking was defined as smoking 100 or more cigarettes and currently smoking every day or some days. Alcohol consumption was determined by self-reported history of alcohol intake, the type of alcoholic beverages consumed, frequency of alcohol consumption, and average quantity consumed per day per week. Current drinking was defined as having had at least one alcoholic beverage in the 12 months prior interviews. Systolic and diastolic blood pressure were measured on the right arm with an appropriately sized cuff using a Baum mercury sphygmomanometer (WA Baum Co) after the participant had been resting in a seated position for 5 minutes. Anthropometric measures were obtained from each participant wearing light clothing and no shoes. Body mass index (BMI) was calculated as body weight in kilograms divided by the square of height in meters (kg/m^2^). Waist-to-hip ratio was calculated as waist circumference divided by hip circumference. Hypertension was defined as blood pressure ≥140/90 mm Hg or current use of antihypertensive medications. [41] According to the American Diabetes Association criteria, diabetes was defined as fasting plasma glucose ≥7.0 mmol/L (126 mg/dl) or currently receiving insulin or oral hyperglycemic treatment [42].

### Statistical analyses

The association of LTL (continuous variable) with traditional coronary risk factors was assessed by calculating partial correlation coefficients, adjusting for age at baseline. All statistical analyses were done using SAS 9.3 (SAS Institute, Inc., Cary, North Carolina).

### Prospective association of LTL with incidence and progression of carotid plaque

The distribution of plaque-free time according to LTL tertiles was estimated by the Kaplan-Meier method. [43] Cox's proportional hazard model was used to assess the association of LTL with development of carotid plaque, adjusting for covariates at baseline [44].

We investigated whether LTL at baseline predicts future onset of carotid plaque and its progression using multivariate survival analysis, in which time (in years) to incident plaque (yes/no) or carotid plaque progression (yes/no) was the dependent variable and LTL (either continuous or in tertiles) was the independent variable, adjusting for baseline covariates described below. In this study, we are interested in both the occurrence of atherosclerotic plaque at the end of study follow-up and plaque-free time in atherosclerosis development. For this purpose, we used Cox proportional hazards models to capture both the “time” and the “event” components related to plaque development. Family relatedness was controlled by the shared frailty model implemented in PROC PHREG. We used the clinical examination date that plaque incidence or progression was identified as the date of diagnosis; otherwise follow-up was censored for participants who remained stable or free of carotid plaque at the end of follow-up.

### Sensitivity analyses

Although we know that plaque onset or its progression took place between the two clinical visits, the exact time of their occurrences was not directly observed in present study. To examine whether and how such interval-censored data influence our results, we performed sensitivity analyses using PROC LIFEREG, which fits a Weibull distribution to interval-censored lifetime data by maximum likelihood estimation of distribution parameters [45]. In this procedure, coefficients describe the log of hazard function using time minus a constant for subjects with “event”, thus it is different from coefficients obtained from the PHREG procedure in proportional hazard model [45].

To examine the impact of potential confounders on our results, we constructed a series of hierarchical models: Model 1 adjusted for age at baseline, sex, and study center; Model 2 additionally adjusted for lifestyle factors including BMI, current smoking and alcohol drinking; Model 3 further adjusted for diabetes status, systolic blood pressure, low-density lipoprotein cholesterol and high-density lipoprotein cholesterol, and estimated glomerular filtration rate (eGFR). Multivariate-adjusted hazard ratios (HRs) and 95% confidence intervals (CIs) were calculated for each model.

Diabetes and hypertension are independent risk factors for atherosclerotic CVD. [46] To examine their potential impact on the association of LTL with development of carotid atherosclerosis, we conducted sensitivity analyses by first excluding participants with prevalent diabetes (n=586), and then further excluding subjects with hypertension (n=484) at baseline. To *examine the robustness* of our findings, we also categorized telomere data into quartiles.

## SUPPLEMENTAL FIGURES AND TABLES


