# Supplementary material for: Short leukocyte telomere length predicts incidence and progression of carotid atherosclerosis in American Indians: The Strong Heart Family Study
Source: Aging (Albany NY). 2014 May 28;6(5):414–27. doi: 10.18632/aging.100671 (PMC4069268; doi:10.18632/aging.100671)
Supplement: Supplementary file 1 [file aging-06-414-s001.pdf]

SUPPLEMENTARY MATERIALS

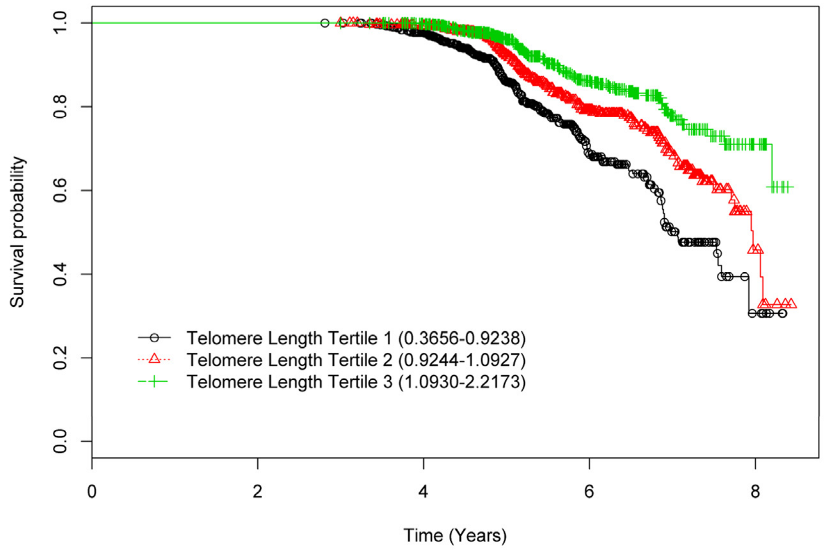

Figure S1. Kaplan-Meier plots for survival function of incident carotid plaque in 2,091 American Indians free of prevalent CVD and carotid plaque at baseline.
